# Rapid identification and prognosis evaluation by dual-phase computed tomography angiography for stroke patients with a large ischemic region in the anterior circulation treated with endovascular thrombectomy

**DOI:** 10.3389/fneur.2024.1402003

**Published:** 2024-05-21

**Authors:** Yajun E, Huigang Jiang, Weifei Yu, Weiwei Chen, Hongfei He

**Affiliations:** ^1^Department of Neurology, Yiwu Central Hospital, Yiwu, China; ^2^Wenzhou Medical University, Zhejiang, China

**Keywords:** stroke, thrombectomy, prognosis evaluation, dual-phase CTA, collateral circulation, collateral grading

## Abstract

**Purpose:**

To investigate the value of dual-phase head-and-neck computed tomography angiography (CTA) in assessing advantages and risks associated with mechanical thrombectomy for stroke with a large ischemic region in the anterior circulation within 6 h of onset.

**Methods:**

We retrospectively analyzed the data of patients with acute occlusion of the internal carotid artery or middle cerebral artery-M1 segment. Baseline dual-phase CTA was performed for collateral grading using the 4-point visual collateral score (0, 0% filling; 1, >0% and ≤50% filling; 2, >50 and <100% filling; 3, 100% filling). The rates of modified Rankin score (MRS) ≤ 3 at 90 days, any intracranial hemorrhage (ICH) within 48 h, malignant cerebral edema within 24 h, and all-cause 90-day mortality were analyzed.

**Results:**

Among the 69 study patients, 15, 26, 17, and 11 patients had collateral grades of 0, 1, 2, and 3, respectively. At 90 days, the MRS was ≤3 in 0, 8.33, 29.41, and 36.36% of patients with grades 0, 1, 2, and 3, respectively. ICH incidence was 73.33, 57.69, 29.41, and 18.18% for grades 0, 1, 2, and 3, respectively, while the incidence of malignant brain edema was 100, 76.92, 35.29, and 0%, respectively. All-cause 90-day mortality was 53.33% for grade 0 and 30.77% for grade 1; no deaths occurred at grades 2 and 3.

**Conclusion:**

Collateral grading based on dual-phase CTA enables simple and rapid preoperative evaluation prior to mechanical thrombectomy for acute anterior-circulation stroke with a large ischemic focus, particularly for patients presenting within the 6-h time window.

## Introduction

Advances in equipment and operative techniques have led to recanalization rates exceeding 80% after mechanical thrombectomy for the treatment of acute anterior-circulation large-vessel occlusion (1, 2). Nonetheless, even when the target vessel is successfully opened within the 6-h time window, the rate of favorable prognosis (defined as functional independence within 90 days after the operation) remains around 40–50% (2). This poor outcome may stem from multiple factors, with the primary contributor being intracranial large-vessel occlusion with poor collateral circulation. For these patients, the large ischemic area following intracranial target-vessel occlusion rapidly progresses to infarction, rendering intravascular therapy ineffective and potentially increasing treatment-related damage (3). Recent trials have demonstrated that patients with extensive cerebral infarctions experience improved functional outcomes with endovascular therapy compared with medical care alone, but they also experience more intracranial hemorrhages (ICHs) and other complications (4–6). Consequently, the early and rapid identification and prognostic evaluation of stroke patients with a large ischemic region are of utmost importance. Given the time-dependent changes in density on non-contrast computed tomography (NCCT) following cerebral ischemia and infarction, the Alberta Stroke Program Early Computed Tomography Score (ASPECTS) based on NCCT presents limitations for ultra-early infarct evaluation. The sensitivity of computed tomography angiography source images (CTA-SI) for detecting infarct foci is significantly higher than that of NCCT (7, 8), though single-phase CTA alone is insufficient for both primary occlusion detection and collateral capacity estimation (9–11).

Therefore, the objective of this study was to determine the value of dual-phase head-and-neck CTA in assessing the advantages and risks associated with mechanical thrombectomy for stroke patients who have a large ischemic region in the anterior circulation and present within 6 h of onset.

## Methods

### Selection criteria

We retrospectively analyzed the imaging and clinical data of patients with large-vessel occlusion involving the anterior circulation who underwent emergency interventional thrombectomy at the Affiliated Yiwu Hospital of Wenzhou Medical University, between January 1, 2020 and January 1, 2023.

Patients were eligible for inclusion in this study if they satisfied the following criteria: (1) patients aged 18 years or older; (2) patients with acute ischemic stroke and a score of at least 6 on the National Institutes of Health Stroke Scale (NIHSS) at admission (NIHSS scores range from 0 to 42, with higher scores indicating greater neurologic deficit); (3) patients with a score of 0 or 1 on the modified Rankin scale (mRS) before the onset of stroke (mRS scores range from 0 to 6, with 0 indicating no disability, 1 indicating no clinically significant disability, 2 indicating slight disability, 3 indicating moderate disability but able to walk unassisted, 4 indicating moderately severe disability, 5 indicating severe disability, and 6 indicating death); (4) patients with an occlusion of the distal internal carotid artery (ICA) or M1 segment of the middle cerebral artery (MCA-M1); and (5) patients with a baseline CTA-SI ASPECTS <6 (if thrombectomy was performed with strategies such as stent retriever, contact aspiration, or combined contact aspiration and stent retriever; if femoral-artery puncture was performed within 6 h after stroke onset; if endovascular therapy could be initiated within 60 min after imaging examination; if the time from femoral-artery puncture to vessel recanalization was less than 120 min; and if the modified Thrombolysis in Cerebral Ischemia (mTICI) score reached 3 for the anterior circulation after thrombectomy).

Patients were excluded if they had a baseline CTA-SI ASPECTS ≥6; underwent femoral-artery puncture at least 6 h after stroke onset; underwent endovascular therapy at least 60 min after the imaging examination; had a time from femoral-artery puncture to vessel recanalization of 120 min or more; or their mTICI score did not reach 3 for the anterior circulation after thrombectomy. The study was approved by the ethics committee of the Affiliated Yiwu Hospital of Wenzhou Medical University.

### Assessment of collateral status

**Figure 1:**
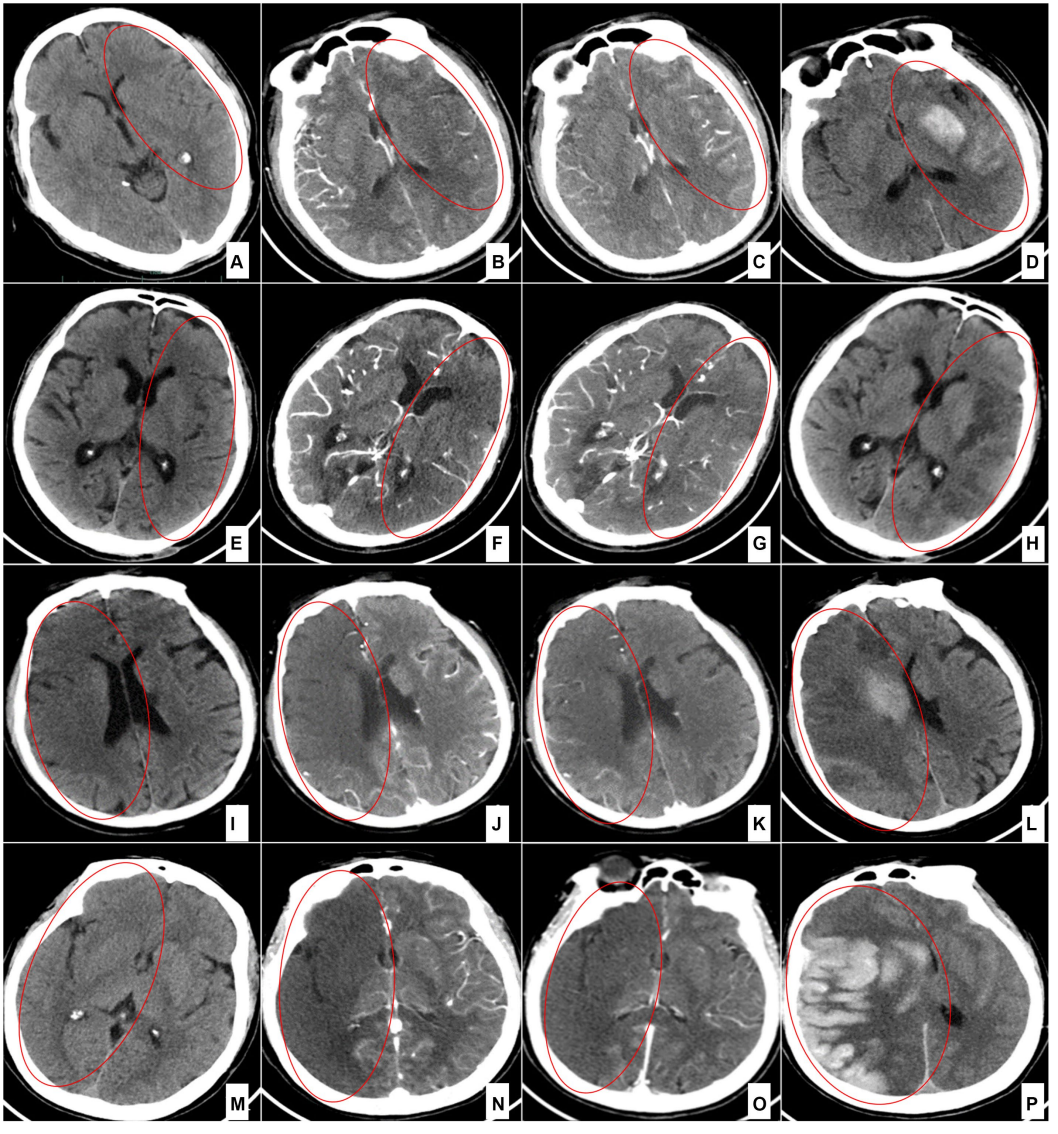
Collateral circulation grading for each category of the 4-point scale. **(A–D)** Grade 3 (100% filling of the occluded territory). A case of left middle cerebral artery occlusion 4 h after onset: preoperative NCCT **(A)**; arterial phase of the CTA source image **(B)**; delayed phase of the CTA source image **(C)**; and NCCT **(D)** 24 h after the operation. **(E–H)** Grade 2 (>50 and < 100% filling of the occluded territory). A case of left middle cerebral artery occlusion 4 h after onset: preoperative NCCT **(E)**; arterial phase of the CTA source image **(F)**; delayed phase of the CTA source image **(G)**; and NCCT **(H)** 24 h after the operation. **(I–L)** Grade 1 (>0% and ≤ 50% filling of the occluded territory). A case of terminal occlusion of the right internal carotid artery 2 h after onset: preoperative NCCT **(I)**; arterial phase of the CTA source image **(J)**; delayed phase of the CTA source image **(K)**; and NCCT **(L)** 8 h after the operation. **(M–P)** Grade 0 (0% filling of the occluded territory). A case of right internal carotid artery occlusion 1 h after onset: preoperative NCCT **(M)**; arterial phase of the CTA source image **(N)**; delayed phase of the CTA source image **(O)**; and NCCT **(P)** 6 h after the operation.

Two neuroradiologists, each with over 5 years of experience, were blinded to the clinical findings and independently evaluated the status of the collateral circulation. In case of any discrepancy between the readers, a third senior neuroradiologist, with over 10 years of experience, was consulted to reach a consensus. The collateral circulation was graded by comparing the dual-phase CTA-SI by using the 4-point visual collateral score (12). Initially, the ischemic range following target-vessel occlusion was identified using the CTA-SI in the early arterial phase. Subsequently, the collateral-circulation status was categorized based on the degree of collateral filling in the same slice of the CTA-SI during the delayed phase (Figure 1). The collateral circulation was graded as follows: grade 0, absent collaterals (0% filling of the occluded vascular territory, Figures 1M–P); grade 1, poor collaterals (>0% and ≤50% filling of the occluded vascular territory, Figures 1I–L); grade 2, moderate collaterals (>50 and <100% filling of the occluded vascular territory, Figures 1E–H); and grade 3, good collaterals (100% filling of the occluded vascular territory, Figures 1A–D). All available slices were utilized to assess the collateral status of the target vessel. If different slices demonstrated varying collateral capacities, the average collateral score across all slices was calculated.

### Clinical and imaging parameters

The following baseline data of the patients were collected: age, sex, history of hypertension, history of diabetes mellitus, history of atrial fibrillation, clinical localization, NIHSS score, occlusion site, CTA-SI ASPECTS, bridging venous thrombolysis, imaging to femoral-artery puncture time, and puncture to vascular recanalization time. The mRS was employed for outcome analysis, and mRS ≤ 3 at 90 days was considered a favorable outcome. The primary safety outcome was any ICH within 48 h after target vessel recanalization. Other safety outcomes included all-cause 90-day mortality and malignant cerebral edema (defined as brain edema with a significant space-occupying effect leading to progressive neurological deterioration, evolving into a malignant state of brain hernia or death, and requiring decompressive craniectomy within 24 h).

### Image acquisition

NCCT and CTA scans were acquired using a standardized protocol on the same multi-section scanner (Optima CT680, 64-slice; General Electric Healthcare, Milwaukee, WI, United States). Axial CT scans were initially obtained for all patients at 120 kV and 60 mA, with 5-mm-thin sections. CTA was subsequently performed, with scanning from the aortic arch to the vertex using a helical scan technique. The CTA acquisitions were obtained after a single intravenous bolus of 90–120 mL of nonionic contrast medium into an antecubital vein at a rate of 3–5 mL/s. Arterial-phase (first-phase) imaging was auto-triggered upon the appearance of contrast medium in the ascending aorta (at 120 HU), followed by delayed-phase imaging immediately after the completion of the first scan. The CTA-SI were reconstructed in the axial plane with 0.625-mm thickness and 0.625-mm intervals as well as with 5-mm thickness and 5-mm intervals. The latter reconstruction was used to evaluate the collateral circulation.

### Statistical analysis

Statistical analysis was conducted using SPSS version 26.0 software (IBM SPSS Inc., Chicago, IL, United States). Continuous variables were represented as means with standard deviations (SDs), while categorical data were presented as counts and percentages. One-way analysis of variance and the Pearson chi-squared test were employed to compare measurement data and categorical data, respectively, among multiple groups, and statistically significant differences were indicated by *p* values of <0.05. Between-group comparisons of clinical and safety outcomes were made using the Fisher exact probability test or Pearson chi-squared test with subsequent Bonferroni correction, and a corrected *p* value of < 0.05 after the Bonferroni adjustment was considered statistically significant.

## Results

A total of 69 patients with anterior-circulation intracranial large-vessel occlusion were included in this study. The distribution of the collateral-circulation status was as follows (Table 1): grade 0 (21.7%, *n* = 15), grade 1 (37.7%, *n* = 26), grade 2 (24.6%, *n* = 17), and grade 3 (15.9%, *n* = 11). The baseline characteristics (Table 1) were evenly distributed across the 4 collateral-circulation grade groups, except for trends in age (*p* = 0.004), sex (*p* = 0.012), history of hypertension (*p* = 0.020), baseline NIHSS score (*p* = 0.011), and occlusion site (*p* = 0.001).

**Table 1:** Clinical and imaging characteristics at the baseline.

Variable	Grade 0 (*n* = 15)	Grade 1 (*n* = 26)	Grade 2 (*n* = 17)	Grade 3 (*n* = 11)	*F*/χ^2^	*p*
Age (y)	72.21 ± 10.14	65.32 ± 7.83	71.32 ± 10.31	59.32 ± 12.83	4.933	0.004
Male sex [*n* (%)]	11 (73.33)	9 (34.62)	12 (70.59)	3 (27.27)	10.871	0.012
History of hypertension [*n* (%)]	11 (73.33)	12 (46.15)	15 (88.24)	5 (45.45)	9.863	0.020
History of diabetes mellitus [*n* (%)]	8 (53.33)	17 (65.38)	11 (64.71)	7 (63.64)	0.669	0.880
Atrial fibrillation [*n* (%)]	10 (66.67)	18 (69.23)	12 (70.59)	5 (45.45)	2.309	0.511
Clinical localization: left hemisphere [*n* (%)]	10 (66.67)	16 (61.54)	13 (76.47)	6 (54.55)	1.670	0.644
Baseline NIHSS score	24.13 ± 3.52	21.83 ± 2.89	19.83 ± 4.61	21.79 ± 2.58	4.048	0.011
Occlusion site: ICA-T [*n* (%)]	12 (80)	18 (69.23)	5 (29.41)	2 (18.18)	16.306	0.001
CTA-SI ASPECTS	3.38 ± 0.83	3.21 ± 0.56	3.37 ± 1.07	3.21 ± 0.92	0.228	0.877
Treatment with IV alteplase [*n* (%)]	11 (73.33)	19 (73.08)	12 (70.59)	8 (72.73)	0.201	1.000
IPT	38.46 ± 9.01	40.46 ± 6.37	35.67 ± 10.02	35.29 ± 9.36	1.560	0. 208
PRT	58.23 ± 16.32	65.17 ± 19.76	61.23 ± 22.87	75.63 ± 21.08	1.766	0.162

NIHSS, National Institutes of Health Stroke Scale; ICA-T, internal carotid artery terminus; CTA, computed tomography angiography; CTA-SI, CTA source images; ASPECTS, Alberta Stroke Program Early CT Score; IV, intravenous; IPT, imaging to puncture time; PRT, puncture to recanalization time.

**Figure 2:**
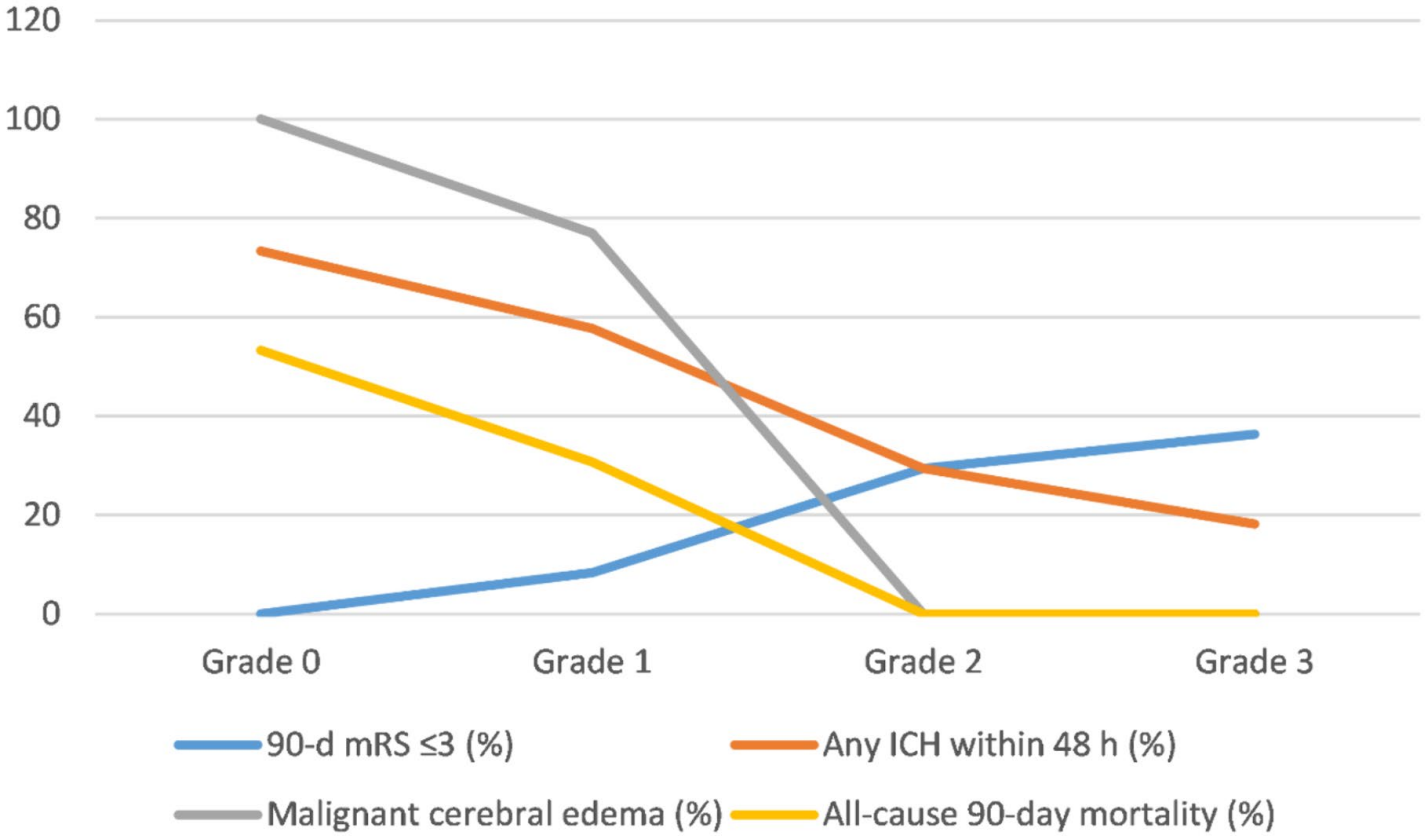
Trends of clinical and safety outcomes after the operation stratified by collateral circulation grade. mRS, modified Rankin score; ICH, intracranial hemorrhage.

The rates of favorable outcomes (mRS ≤ 3) at 90 days were 0, 8.33, 29.41, and 36.36% in the grade 0, 1, 2, and 3 groups, respectively (Table 2). Although the rate of favorable outcomes increased with increasing collateral-circulation grade, no statistically significant differences were found between the 4 grades (*p* > 0.05/6 = 0.0083; Table 2; Figure 2).

**Table 2:** Clinical and safety outcomes.

Collateral grade	90-d mRS ≤ 3 (%)	Any ICH within 48 h (%)	Malignant cerebral edema (%)	All-cause 90-day mortality
Grade 0	0 (0/15)	73.33 (11/15)	100 (15/15)	53.33 (8/15)
Grade 1	8.33 (2/26)	57.69 (15/26)	76.92 (20/26)	30.77 (8/26)
Grade 2	29.41 (5/17)	29.41 (5/17)	35.29^*#^ (6/17)	0^*^ (0/17)
Grade 3	36.36 (4/11)	18.18 (2/11)	0^*#^ (0/11)	0^*^ (0/11)

mRS, modified Rankin score; ICH, intracranial hemorrhage.

Group comparisons were corrected by Bonferroni adjustment, and *p* < 0.05/6 = 0.0083 was considered significant.

* and # indicate a statistically significant difference compared to grades 0 and 1, respectively.

The incidence of any ICH within 48 h was 73.33, 57.69, 29.41, and 18.18% in the grade 0, 1, 2, and 3 groups, respectively (Table 2). Despite the decrease in the incidence of any ICH with increasing collateral-circulation grade, no statistically significant differences were found between the grades (*p* > 0.05/6 = 0.0083; Table 2; Figure 2).

The incidence of malignant brain edema within 24 h was 100, 76.92, 35.29, and 0% in the grade 0, 1, 2, and 3 groups, respectively (Table 2). The incidence of malignant brain edema was significantly lower for grades 2 and 3 than for grades 0 and 1, respectively (*p* < 0.05/6 = 0.0083; Table 2). No significant differences were found between grades 0 and 1 as well as between grades 3 and 4 (*p* > 0.05/6 = 0.0083; Table 2; Figure 2).

The all-cause 90-day mortality rate was 53.33% in the grade 0 group and 30.77% in the grade 1 group; no deaths occurred within 90 days in the grade 2 and 3 groups (Table 2). The all-cause 90-day mortality was significantly lower for grades 2 and 3 than for grade 0, respectively (*p* < 0.05/6 = 0.0083; Table 2). No statistically significant differences were found between the other groups (*p* > 0.05/6 = 0.0083; Table 2; Figure 2).

## Discussion

Among stroke patients who present within the 6-h routine time window, the presence of a large ischemic area is an important factor that can reduce the rate of good prognosis after mechanical thrombectomy. Hence, the ultra-rapid identification and evaluation of stroke patients with a large ischemic region and the estimation of the safety and prognosis of thrombectomy in these patients have crucial clinical implications. With the integration of artificial intelligence (AI) and imaging technology, some automated processing software, such as RAPID^®^ (Rapid Processing of Perfusion and Diffusion), can accurately measure the infarct core and penumbra based on CT perfusion imaging or magnetic resonance−diffusion-weighted imaging (13). However, current diagnostic and treatment guidelines do not recommend overly complex imaging examinations for patients with intracranial large-vessel occlusion who present within 6 h of stroke onset; instead, head NCCT and head-and-neck CTA are often the first choices for the evaluation of these patients prior to thrombectomy (14).

**Figure 3:**
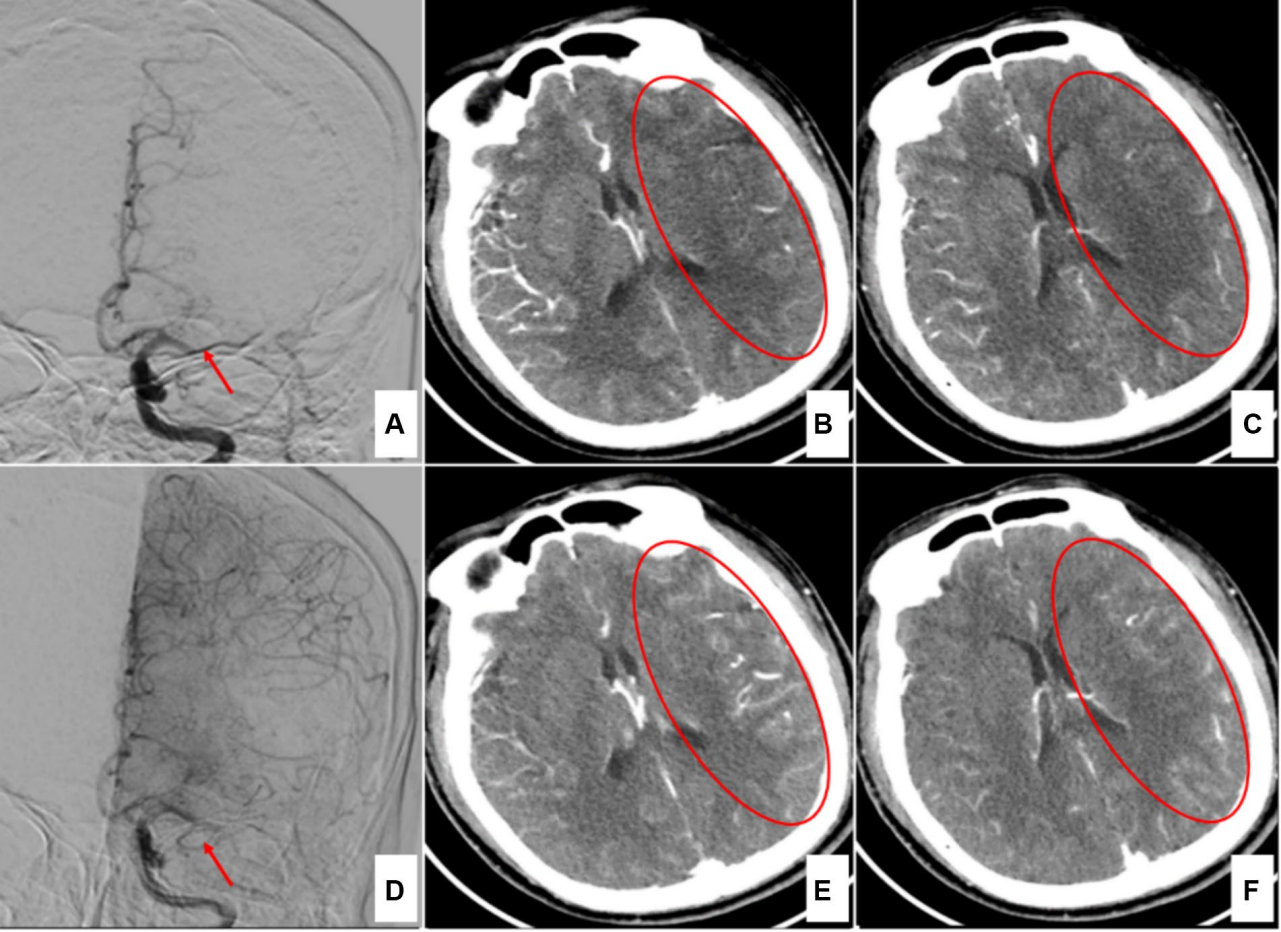
A case of left middle cerebral artery occlusion 4 h after onset. **(A)** Digital subtraction angiography (DSA) in the early arterial phase indicates left middle cerebral artery occlusion (red arrow). **(B,C)** Arterial-phase CTA source images show the ischemic region in the left temporal lobe (red circles). **(D)** DSA in the late arterial phase shows compensatory vessels of the anterior cerebral artery (red arrow). **(E,F)** Delayed-phase CTA source images indicate that the ischemic region has been filled by the compensatory vessels (red circles).

The density changes in ischemic infarcts in brain tissue on NCCT are gradual and time-dependent (15, 16). Some patients in this study arrived at the hospital shortly after stroke onset, resulting in minimal detectable abnormalities on preoperative head NCCT (Figure 1M). This finding further confirmed that the NCCT ASPECTS has certain limitations in recognizing ultra-early massive cerebral infarction. Research has shown that CTA-SI ASPECTS is significantly superior to NCCT ASPECTS for the preoperative evaluation of patients with acute ischemic stroke prior to intravascular treatment (7, 8). Some researchers have also assessed collateral circulation by observing the degree of collateral-vessel filling in the blood supply area of the target occluded vessels on single-phase CTA-SI (17–19). Since the potential compensatory blood supply after the occlusion of the distal ICA or MCA-M1 is often via the leptomeningeal pathway, and since peak collateral flow occurs after the arterial phase, delayed-phase CTA-SI could be more advantageous for estimating collateral circulation (Figure 3). The dual-phase CTA employed in this study added a delayed-phase scan based on conventional head-and-neck CTA, enabling more accurate assessment of the ischemic range and collateral circulation (Figure 3).

Collateral circulation is an essential determinant of clinical outcomes after acute intracranial large-vessel occlusion (20, 21). Poor collateral status is associated with larger follow-up infarct volumes, increased mortality, and unfavorable functional prognosis. Among the patients included in this study, those with collateral circulation grades 2 and 3 had a lower incidence of ICH, malignant brain edema, and mortality than patients with grades 0 and 1, which is consistent with previous studies (22). Considering the inclusion of patients with anterior-circulation large-vessel occlusion with large ischemic lesions (baseline CTA-SI ASPECTS <6), this study defined mRS ≤ 3 (instead of mRS ≤ 2) at 90 days after thrombectomy as a favorable outcome. The incidence rates of favorable outcomes at 90 days in patients with collateral circulation grades 0 and 1 were 0 and 8.33%, respectively, indicating that even though the target vessel was successfully opened in a short period of time, the original large ischemic area quickly transformed into an infarct. Therefore, the treatment time window for these patients may be much less than 6 h. For patients with better collateral circulation (grades 2 and 3), the incidence rates of favorable outcomes at 90 days were only 29.41 and 36.36%, respectively. This finding further demonstrates the importance of minimizing preoperative delay for patients with anterior-circulation large-vessel occlusion accompanied by large ischemic lesions who present within 6 h of onset.

In recent years, the use of perfusion imaging techniques has been expanding to more and more hospitals. These techniques have improved prognostication in acute ischemic stroke and enabled the identification of patients with treatment targets well beyond the conventional time window for intravenous thrombolysis or endovascular treatment or both (23–26). However, perfusion imaging techniques do not directly provide information on collateral circulation, and to some extent increase the examination time, leading to delayed treatment. This is particularly not recommended for acute ischemic stroke patients with poor collateral circulation who present within 6 h of onset (14). Therefore, this study employed dual-phase CTA for the preoperative workup of stroke patients with a large ischemic region in the anterior circulation who presented within 6 h of onset. Dual-phase CTA may have some advantages in this population, especially in terms of not requiring too much additional examination time.

It is worth noting that compared to previous research (4), this study has a relatively high ICH rate, especially in the grade 0 and 1 groups. This is attributable to the rapid progression of large ischemic lesions with poor collateral circulation to infarction, leading to an increased likelihood of reperfusion hemorrhage after target vessel recanalization. The high ICH rate is also attributable to the relatively high rate of intravenous thrombolysis bridging endovascular therapy in the patients included in this study (Table 1). This study included patients who were screened prior to mechanical thrombectomy based on the CTA-SI ASPECTS primarily identifying large ischemic lesions by using the filling defect area on the arterial-phase CTA-SI images. Such lesions often do not show severe or extensive density changes on NCCT during the ultra-early infarct stage or even within 4.5 h of onset. In fact, this is one of the key points that this research revealed: neither NCCT nor single arterial-phase CTA-SI could accurately reflect the infarct core; NCCT might underestimate the core, while CTA-SI might overestimate it. However, considering that this study was single-center study with a small sample size, a clear conclusion could not be drawn yet, and further in-depth research is needed in the future.

## Limitations

First, the acquisition of arterial- and delayed-phase data by head-and-neck CTA is influenced by the CT scanning equipment, and the optimal display time of the collateral circulation is affected by the location of the large-vessel occlusion, the variability of contrast injection, the compensatory capacity, and the extent of collateral vessels. Consequently, the selection of CTA-data acquisition time points in this study requires further optimization (27). Second, image resolution may affect the accuracy of the assessment of the collateral circulation status, and further optimization and adjustments to the image resolution are needed (28). Third, collateral grading necessitates manual judgment, which is inevitably influenced by the subjectivity of the evaluator and can vary based on the experience of the radiologist. Finally, this study is a retrospective, single-center study, and the small sample size limited the ability to draw definitive and causal conclusions.

## Conclusion

Dual-phase head-and-neck CTA could more comprehensively display the ischemic range and local collateral status after intracranial large-vessel occlusion. Collateral grading based on dual-phase CTA offers a simple and rapid method for the preoperative evaluation of patients with acute anterior-circulation stroke with a large area of ischemic focus prior to mechanical thrombectomy. This is especially important for patients who present within the 6-h time window.

## Data availability statement

The original contributions presented in the study are included in the article/supplementary material, further inquiries can be directed to the corresponding author.

## Ethics statement

The studies involving humans were approved by the ethics committee of the Affiliated Yiwu Hospital of Wenzhou Medical University (2023-IRB-019). The studies were conducted in accordance with the local legislation and institutional requirements. The participants provided their written informed consent to participate in this study. Written informed consent was obtained from the individual(s) for the publication of any potentially identifiable images or data included in this article.

## Author contributions

YE: Conceptualization, Data curation, Formal analysis, Funding acquisition, Investigation, Methodology, Project administration, Resources, Software, Supervision, Validation, Visualization, Writing – original draft, Writing – review & editing. HJ: Data curation, Formal analysis, Investigation, Methodology, Software, Writing – review & editing. WY: Data curation, Investigation, Methodology, Software, Supervision, Validation, Visualization, Writing – original draft. WC: Data curation, Investigation, Methodology, Software, Writing – original draft, Writing – review & editing. HH: Data curation, Formal analysis, Software, Writing – original draft, Investigation.

## References

[1] Goyal M Menon BK van Zwam WH Dippel DWJ Mitchell PJ Demchuk AM. Endovascular thrombectomy after large-vessel ischaemic stroke: a meta-analysis of individual patient data from five randomised trials. Lancet. (2016) 387:1723–31. doi: 10.1016/S0140-6736(16)00163-X, PMID: 26898852

[2] Tong X Wang Y Fiehler J Bauer CT Jia B Zhang X. Thrombectomy versus combined thrombolysis and Thrombectomy in patients with acute stroke: a matched-control study. Stroke. (2021) 52:1589–600. doi: 10.1161/STROKEAHA.120.031599 33657849

[3] Goyal M Ospel JM Menon B Almekhlafi M Jayaraman M Fiehler J. Challenging the ischemic Core concept in acute ischemic stroke imaging. Stroke. (2020) 51:3147–55. doi: 10.1161/STROKEAHA.120.030620, PMID: 32933417

[4] Huo X Ma G Tong X Zhang X Pan Y Nguyen TN. Trial of endovascular therapy for acute ischemic stroke with large infarct. N Engl J Med. (2023) 388:1272–83. doi: 10.1056/NEJMoa2213379 36762852

[5] Yoshimura S Sakai N Yamagami H Uchida K Beppu M Toyoda K. Endovascular therapy for acute stroke with a large ischemic region. N Engl J Med. (2022) 386:1303–13. doi: 10.1056/NEJMoa2118191 35138767

[6] Sarraj A Hassan AE Abraham MG Ortega-Gutierrez S Kasner SE Hussain MS. Trial of endovascular Thrombectomy for large ischemic strokes. N Engl J Med. (2023) 388:1259–71. doi: 10.1056/NEJMoa2214403 36762865

[7] Park JS Lee JM Kwak HS Chung GH. Predictive value of CT angiography source image ASPECTS in patients with anterior circulation acute ischemic stroke after endovascular treatment: ultimate infarct size and clinical outcome. J Neurointerv Surg. (2019) 11:342–6. doi: 10.1136/neurintsurg-2018-014359, PMID: 30472673

[8] Sallustio F Motta C Pizzuto S Diomedi M Rizzato B Panella M. CT angiography ASPECTS predicts outcome much better than noncontrast CT in patients with stroke treated Endovascularly. AJNR Am J Neuroradiol. (2017) 38:1569–73. doi: 10.3174/ajnr.A5264, PMID: 28619833 PMC7960404

[9] Wiegers EJA Mulder MJHL Jansen IGH Venema E Compagne KCJ Berkhemer OA. Clinical and imaging determinants of collateral status in patients with acute ischemic stroke in MR CLEAN trial and registry. Stroke. (2020) 51:1493–502. doi: 10.1161/STROKEAHA.119.027483 32279619

[10] Menon BK d'Esterre CD Qazi EM Almekhlafi M Hahn L Demchuk AM. Multiphase CT angiography: a new tool for the imaging triage of patients with acute ischemic stroke. Radiology. (2015) 275:510–20. doi: 10.1148/radiol.15142256, PMID: 25633505

[11] Morelli N Rota E Immovilli P Marchesi G Guidetti D Michieletti E. Dual-phase 16 slice CT angiography in stroke imaging: a poor man's multiphase study? Acta Neurol Belg. (2019) 119:187–92. doi: 10.1007/s13760-018-1019-4, PMID: 30196370

[12] Tan IYL Demchuk AM Hopyan J Zhang L Gladstone D Wong K. CT angiography clot burden score and collateral score: correlation with clinical and radiologic outcomes in acute middle cerebral artery infarct. AJNR Am J Neuroradiol. (2009) 30:525–31. doi: 10.3174/ajnr.A1408, PMID: 19147716 PMC7051470

[13] Nogueira RG Jadhav AP Haussen DC Bonafe A Budzik RF Bhuva P. Thrombectomy 6 to 24 hours after stroke with a mismatch between deficit and infarct. Randomized controlled trial. N Engl J Med. (2018) 378:11–21. doi: 10.1056/NEJMoa1706442, PMID: 29129157

[14] Powers WJ Rabinstein AA Ackerson T Adeoye OM Bambakidis NC Becker K. Guidelines for the early Management of Patients with Acute Ischemic Stroke: 2019 update to the 2018 guidelines for the early Management of Acute Ischemic Stroke: a guideline for healthcare professionals from the American Heart Association/American Stroke Association. Stroke. (2019) 50:e344–418. doi: 10.1161/STR.0000000000000211, PMID: 31662037

[15] Camargo EC Furie KL Singhal AB Roccatagliata L Cunnane ME Halpern EF. Acute brain infarct: detection and delineation with CT angiographic source images versus nonenhanced CT scans. Radiology. (2007) 244:541–8. doi: 10.1148/radiol.2442061028, PMID: 17581888

[16] Schramm P Schellinger PD Fiebach JB Heiland S Jansen O Knauth M. Comparison of CT and CT angiography source images with diffusion-weighted imaging in patients with acute stroke within 6 hours after onset. Stroke. (2002) 33:2426–32. doi: 10.1161/01.str.0000032244.03134.37, PMID: 12364733

[17] Berkhemer OA Jansen IG Beumer D Fransen PSS van den Berg LA Yoo AJ. Collateral status on baseline computed tomographic angiography and intra-arterial treatment effect in patients with proximal anterior circulation stroke. Stroke. (2016) 47:768–76. doi: 10.1161/STROKEAHA.115.011788, PMID: 26903582

[18] Boers AMM Sales Barros R Jansen IGH Berkhemer OA Beenen LFM Menon BK. Value of quantitative collateral scoring on CT angiography in patients with acute ischemic stroke. AJNR Am J Neuroradiol. (2018) 39:1074–82. doi: 10.3174/ajnr.A5623, PMID: 29674417 PMC7410629

[19] Wolff L Uniken Venema SM Luijten SPR Hofmeijer J Martens JM Bernsen MLE. Diagnostic performance of an algorithm for automated collateral scoring on computed tomography angiography. Eur Radiol. (2022) 32:5711–8. doi: 10.1007/s00330-022-08627-4 35244761 PMC9279191

[20] Park JS Kwak HS Chung GH Hwang S. The prognostic value of CT-angiographic parameters after reperfusion therapy in acute ischemic stroke patients with internal carotid artery terminus occlusion: leptomeningeal collateral status and clot burden score. J Stroke Cerebrovasc Dis. (2018) 27:2797–803. doi: 10.1016/j.jstrokecerebrovasdis.2018.06.010, PMID: 30064866

[21] Lima FO Furie KL Silva GS Lev MH Camargo ECS Singhal AB. The pattern of leptomeningeal collaterals on CT angiography is a strong predictor of long-term functional outcome in stroke patients with large vessel intracranial occlusion. Stroke. (2010) 41:2316–22. doi: 10.1161/STROKEAHA.110.592303, PMID: 20829514 PMC4939434

[22] Leng X Fang H Leung TWH Mao C Miao Z Liu L. Impact of collaterals on the efficacy and safety of endovascular treatment in acute ischaemic stroke: a systematic review and meta-analysis. J Neurol Neurosurg Psychiatry. (2016) 87:537–44. doi: 10.1136/jnnp-2015-310965, PMID: 26063928

[23] Jovin TG Nogueira RG Lansberg MG Demchuk AM Martins SO Mocco J. Thrombectomy for anterior circulation stroke beyond 6 h from time last known well (AURORA): a systematic review and individual patient data meta-analysis. Lancet. (2022) 399:249–58. doi: 10.1016/S0140-6736(21)01341-6 34774198

[24] Desai SM Haussen DC Aghaebrahim A Al-Bayati AR Santos R Nogueira RG. Thrombectomy 24 hours after stroke: beyond DAWN. J Neurointerv Surg. (2018) 10:1039–42. doi: 10.1136/neurintsurg-2018-013923, PMID: 29807887

[25] Ma H Campbell BCV Parsons MW Churilov L Levi CR Hsu C. Thrombolysis guided by perfusion imaging up to 9 hours after onset of stroke. N Engl J Med. (2019) 380:1795–803. doi: 10.1056/NEJMoa1813046, PMID: 31067369

[26] Campbell BCV Ma H Ringleb PA Parsons MW Churilov L Bendszus M. Extending thrombolysis to 4·5-9 h and wake-up stroke using perfusion imaging: a systematic review and meta-analysis of individual patient data. Lancet. (2019) 394:139–47. doi: 10.1016/S0140-6736(19)31053-0, PMID: 31128925

[27] Pulli B Schaefer PW Hakimelahi R Chaudhry ZA Lev MH Hirsch JA. Acute ischemic stroke: infarct core estimation on CT angiography source images depends on CT angiography protocol. Radiology. (2012) 262:593–604. doi: 10.1148/radiol.11110896, PMID: 22187626 PMC3267077

[28] Versaci M Angiulli G Crucitti P Carlo DD Laganà F Pellicanò D. A fuzzy similarity-based approach to classify numerically simulated and experimentally detected carbon Fiber-reinforced polymer plate defects. Sensors (Basel). (2022) 22:4232. doi: 10.3390/s22114232, PMID: 35684853 PMC9185562

